# Editorial: Assessing the value and cost of Organ Donation and Transplantation (ODT)

**DOI:** 10.3389/fpubh.2024.1388317

**Published:** 2024-03-19

**Authors:** Lara Gitto, Evaldo Favi, Emmanouil Giorgakis, Roberto Cacciola

**Affiliations:** ^1^Dipartimento di Economia, Università Degli Studi di Messina, Messina, Italy; ^2^General Surgery and Kidney Transplantation, Fondazione IRCCS Ca' Granda Ospedale Maggiore Policlinico, Milan, Italy; ^3^Clinical Sciences and Community Health, Università Degli Studi di Milano, Milan, Italy; ^4^Division of Solid Organ Transplantation, Department of Surgery, UAMS Medical Center, Little Rock, AR, United States; ^5^Department of Surgical Sciences, Tor Vergata University, Rome, Italy

**Keywords:** organ donation (topic area), transplantation, health economics, kidney transplantation, living donation, benchmark

Healthcare financing has been defined by the World Health Organization as a “core function of health systems” (1); such a pertinent statement underscores the importance of better understanding the value and costs of Organ Donation and Transplantation (ODT). Although the clinical value of transplantation remains undisputed, the financial value and real costs of the whole process of offering life-saving or life-prolonging treatment to thousands of patients worldwide each year have only rarely been analyzed in-depth and even more rarely contextualized as a unique, multidisciplinary, and interdependent healthcare entity (Leonardis et al.). In particular, the value of the complex activities consisting of several phases, supporting organ donation and retrieval and culminating in the actual transplantation procedure, should also be analyzed against the social and treatment costs of managing patients with End-Stage Organ Failure. Equally important, the economic benefits together with the efficiency of the procedures and their value are not only evident for the patients and their families, but for the whole community; therefore, the definition of the actual “value” should not be limited to a favorable cost-benefit analysis, but should extend to the social aspects of care, which may be very difficult to capture and fully appreciate.

The many steps and procedures involved in the transplant of organs that are immediately life-saving are generally perceived and accounted for as highly expensive for any healthcare service. This is undoubtedly true; however, the actual financial savings produced by the “gold standard” of treatment for eligible patients with End-Stage Kidney Disease (ESKD), that is Kidney Transplantation (KT), can comfortably offset the costs of Organ Donation (OD) services. Therefore, a “Keynesian perspective” of the health economics of KT where savings represent the actual financial resources, may benefit the whole ODT service to the extent that financial self-sufficiency can be achieved (Leonardis et al.).

There are several aspects of ODT practice that will contribute to improving the understanding of the value of the service. It was indicated by Kim et al. that KT is the preferred Renal Replacement Therapy (RRT) for suitable patients, adding that Living Donor Kidneys should be preferred over Deceased Donor kidneys because of their superior quality, which results in improved patient and graft survival. Such considerations extend to the issue of supply and demand in the “economics of transplants.” Boadu et al. outlined in their analysis carried out on different population subgroups in England, that there is an emerging consensus supporting the concept that an increase in living donation could contribute even more than deceased donation to reducing inequalities in organ donation. The machine learning (ML) approach used in the study identified important factors that influence intentions to become a living kidney donor. Support for organ donation, awareness of public campaigns, and younger age were all positively associated with the predicted propensity to become a living donor (Boadu et al.). Zhang et al. also highlighted that organ donation is a prosocial behavior as it is aimed at prolonging the life of the recipient. However, the authors report that the supply-demand ratio of organs in China is highly unbalanced and it should be investigated what hampers organ donation in their country, which is characterized by diverse social representations and perceived barriers to organ donation (Zhang et al.). In this context, the media may be a highly relevant channel for improving organ donation knowledge; in this sense, Gong et al. explored the influence of media use on willingness to donate organs and the factors influencing willingness to donate organs in people with different levels of media use. Their results outlined how high-frequency media users are positively correlated with their willingness to consider organ donation. Hence, it is necessary to formulate personalized and targeted dissemination strategies for health information on organ donation for different media users (Gong et al.). Confirmation of the global need for adequate education also comes from the contribution of Jazienicka-Kiełb et al., who assessed the knowledge of CKD among primary care physicians (PCPs) in Poland. They reported that despite a fairly high level of knowledge among PCPs regarding the causes, risk factors, and progression of CKD, there is still a need for further education and an increase in factual information among this professional group (Jazienicka-Kiełb et al.). The importance of knowledge and education about ODT is also highlighted in the study by Wang et al., which focused on target populations who have undergone solid organ transplant (SOT) to bolster preventative practices in these patients during the Coronavirus Disease 2019 (COVID-19) pandemic. They report that while sufficient levels of knowledge are generally correlated with a higher likelihood of adequate levels of practice, they found that positive attitudes toward transplantation were not correlated with adequate levels of practice in the United states (Wang et al.).

The financial insecurity caused by global instability and the ongoing COVID-19 pandemic caused by SARS-CoV-2 have exposed several weaknesses of the entire healthcare system, with hard-to-estimate, but predictably significant repercussions on the healthcare services routinely offered to worldwide users (Leonardis et al.). Hence, a reliable estimate of the financial value of ODT paired with an optimization strategy is of critical relevance, for both developing and developed countries. Indeed, the substantial savings generated by KT benefit the entire healthcare service. The work of Leonardis et al. on the evaluation of the actual financial benefits generated by KT, offers a novel perspective on the health economics of KT and SOT in general, and developed a specific methodology for the definition of a novel coefficient; the Kidney Transplant Coefficient of Value (KTCoV) which contributes to a reliable estimation of the savings generated by KT activity. It is also maintained that an adequate optimization of the funding process can lead to the financial self-sufficiency of the ODT service.

A more in-depth benchmark analysis of three different ODT programs produced by Cacciola et al. compared the financial resources obtained in the Italian regions of Sicily and Lazio with those obtained in Scotland identifying multiple and interdependent, factors influencing the different levels of KT activity with an estimate of the associated “foregone savings.” Organ donation rates, access to the transplant waiting list, and KT from living donors appear to be the most prominent determinants of the observed different levels of activity. In this light, the Authors suggest replicating the international experience with a comprehensive strategy to be implemented by a “task force” that would successfully address the critical areas of the service to reverse the observed trend and promote the growth of the service (Cacciola et al.).

A constructive governance process is critical to the achievement of positive outcomes for both patients and healthcare commissioners (2); therefore psychosocial aspects of care undoubtedly represent a highly relevant aspect of ODT. In their contribution, Zerbinati et al. investigated the psychosocial factors that frequently occur in kidney transplant recipients and that lead to behavioral changes and reduced therapeutic adherence. Their study showed that somatization and mood disorders may predict costs for hospital admissions and emergency department use and may be risk factors for poor outcomes, including death, in KT (Zerbinati et al.).

In conclusion, the value of ODT extends from the highly successful clinical practice of saving or prolonging the lives of patients with end-stage organ disease, to the positive impact on their families and wider society. Uniquely, the clinical and social benefits are also associated with conspicuous savings that should attract the attention of healthcare commissioners. The traditional fee-for-service funding methodology, in which providers are paid based on the number of healthcare services they deliver does not reflect the needs and value of the whole ODT. Consequently, it can be argued that ODT, because of its complexity and the highly successful practice of SOT, consisting of patient and graft survival rates, would benefit from being funded as an in-hospital service with a comprehensive fee-for-value rather than a compartmentalized fee-for-service funding method [Leonardis et al.; (3)].

## Author contributions

LG: Conceptualization, Project administration, Supervision, Writing – original draft, Writing – review & editing. EF: Data curation, Methodology, Validation, Writing – review & editing. EG: Data curation, Formal analysis, Investigation, Validation, Writing – review & editing. RC: Writing – original draft, Writing – review & editing.
